# Advanced Pedestrian Positioning System to Smartphones and Smartwatches

**DOI:** 10.3390/s16111903

**Published:** 2016-11-11

**Authors:** Alejandro Correa, Estefania Munoz Diaz, Dina Bousdar Ahmed, Antoni Morell, Jose Lopez Vicario

**Affiliations:** 1 Telecommunications and Systems Engineering Department, Universitat Autònoma de Barcelona, Bellaterra 08193, Spain; antoni.morell@uab.cat (A.M.); jose.vicario@uab.cat (J.L.V.); 2 Institute of Communications and Navigation, German Aerospace Center, Oberpfaffenhofen 82234, Germany; estefania.munoz@dlr.de (E.M.D.); dina.bousdarahmed@dlr.de (D.B.A.)

**Keywords:** inertial sensors and systems, smartphone navigation systems, aiding technology for INS, smartwatch, received signal strength

## Abstract

In recent years, there has been an increasing interest in the development of pedestrian navigation systems for satellite-denied scenarios. The popularization of smartphones and smartwatches is an interesting opportunity for reducing the infrastructure cost of the positioning systems. Nowadays, smartphones include inertial sensors that can be used in pedestrian dead-reckoning (PDR) algorithms for the estimation of the user’s position. Both smartphones and smartwatches include WiFi capabilities allowing the computation of the received signal strength (RSS). We develop a new method for the combination of RSS measurements from two different receivers using a Gaussian mixture model. We also analyze the implication of using a WiFi network designed for communication purposes in an indoor positioning system when the designer cannot control the network configuration. In this work, we design a hybrid positioning system that combines inertial measurements, from low-cost inertial sensors embedded in a smartphone, with RSS measurements through an extended Kalman filter. The system has been validated in a real scenario, and results show that our system improves the positioning accuracy of the PDR system thanks to the use of two WiFi receivers. The designed system obtains an accuracy up to 1.4 m in a scenario of 6000 $m^{2}$.

## 1. Introduction

The need for positioning and localization has increased over the decades, relying mostly on GPS to fulfill the task. However, indoor environments, among other scenarios, such as urban canyons, are satellite-denied environments; which makes it infeasible to rely on GPS. To overcome this challenge, research on non-satellite-based solutions has experienced a rapid growth during the last few years. The diversity of solutions is overwhelming. Technology and algorithmic choices condition each system’s strengths and weaknesses, thus making them suitable only for certain applications.

We focus ourselves on mass market applications. The evolution of micro-electromechanical systems (MEMS) in cost, size and quality made it possible for these to be embedded in every smartphone. Nevertheless, the mass market does not end here. We foresee a growth in the demand of smart devices, with smartwatches among them. This implies larger amounts of position-related information available. MEMS accelerometers and gyroscopes comprise an inertial measurement unit (IMU), and their first use in smartphones was to determine the orientation, landscape or portrait, of the device. In recent years, there has been an increasing interest in developing pedestrian dead-reckoning (PDR) algorithms based on an IMU embedded in a smartphone.

There are two types of PDR, the strapdown algorithm and the step-length-and-heading-estimation approach. Standard strapdown algorithms [1] use turn rates to keep track of the IMU orientation, thus being able to subtract the gravity effect from the accelerometer signal. Then, the corrected acceleration is used to compute the position by double integration. The accumulated error in the position due to the integration of current MEMS inertial sensor readings is prohibitively high. Possible solutions are zero-velocity updates (ZUPTs) [2], though these require a foot-mounted IMU to detect the foot stance phase.

The step-length-and-heading-estimation approach sequentially estimates the pedestrian’s position based on a previous computed position and an estimation of both the step length and direction of the walk, also known as heading [3]. A well-known approach to identify steps is to detect changes in the vertical displacement of the pelvis as in [4]. Other options are to find a model based on the acceleration signal in the time domain [5,6] or in the frequency domain [7]. Most recent approaches use a pitch-based model for pocket-mounted IMUs [8].

The problems of inertial positioning systems can be circumvented by the development of hybrid systems that combine the inertial measurements with position estimations from network-based positioning algorithms. These systems estimate the user’s position through the communication of the user with a wireless network (WiFi, LTE, ultra-wide band (UWB), wireless sensor networks (WSN), Bluetooth). Traditionally, these systems have been classified depending on the way the distance between any two nodes of the network is estimated. There are methods based on the time of arrival (ToA), methods based on the angle of arrival (AoA) and methods based on the received signal strength (RSS) [9,10,11]. Nowadays, ToA methods employing UWB signals show encouraging results [12,13]. However, and focusing on our target of mass market applications, RSS-based methods based on WiFi networks are the most suitable option since WiFi networks are deployed all around the world in millions of buildings.

The combination of RSS-based methods and inertial methods can be done in multiple ways. For example, in [14,15], the authors use the RSS to compute the initial location of the user. Other authors use a Kalman filter (KF) to combine the RSS and inertial measurements [16,17,18]. It is also common to employ different fusion algorithms as in [19,20], where an extended Kalman filter (EKF) is used, or in [21], where the authors use a particle filter (PF). As an alternative, digital information of the map of the building can be used to enhance the position estimation [22,23]. Unfortunately, the information of the map is not always available for positioning purposes.

Particularizing for the problem of interest in this work, i.e., indoor positioning systems based on smartphones, we can find related works in the literature as [24], where the authors present an indoor tracking system for underground public transportation based on inertial measurements and information about the route and average time between stops. Other authors estimate the relative position of the smartphone with respect to the user, which is mandatory for the transformation of the inertial measurements from the smartphone coordinate frame to the navigation coordinate frame; in [25], the authors use a least square support vector machine (LS-SVM). However, in order to increase the accuracy of the system, the authors typically predefine the position of the smartphone; an example can be found in [6], where the authors propose a pure inertial-based positioning system specifically designed for smartphones. The main disadvantage of pure inertial systems is that they suffer from the inertial drift, and therefore, the error increases with time. To avoid the effects of the inertial drift, the authors use hybrid solutions that fuse the inertial measurements with RSS measurements. The authors in [26] formulate the fusion of the RSS with the inertial measurements through a hidden Markov model (HMM). Alternatively, in [27], the fusion algorithm used is the sequential Monte Carlo Kalman filter. In [28] the authors combine the inertial measurements with RSS and magnetic fingerprinting using a KF. The accuracy of this system is typically around 3 m. Another approach to improve the accuracy is the simultaneous localization and mapping (SLAM) approach, as in [29,30], but the accuracy is only increased if the path walked by the user forms closed loops. An alternative is the use of information about the floor plan or landmark points (stairs, elevators, doors, etc.) [31,32,33]. This approach increases the accuracy of the system, but cannot be generalized, as the map of the building is not always available.

Nevertheless, a major problem with these kinds of systems when applied to mass market applications is the lack of control over the WiFi network. The design of WiFi networks is traditionally done for communications purposes, and therefore, it introduces problems when used for positioning applications; for example: (i) the lack of measurements from different anchor nodes or (ii) the quality of the RSS measurements for distance estimations due to the density of the nodes of the network. So far, however, there has been little discussion about the effects of the application of positioning systems to a new scenario where the WiFi network is not controlled by the designer of the positioning system. In this work, we identify and evaluate these effects and present a method that ameliorates them by introducing a second WiFi receiver in the system. Particularly, we will use the RSS measurements obtained with a smartphone and a smartwatch. We design a new method for combining RSS measurements from different receivers and inertial measurements from a low-cost IMU embedded in a commercial smartphone placed in the pocket of the user. The result is a pedestrian positioning system that runs in commercial smartphones and smartwatches, and thus, it is suitable for mass market applications. The main contributions of this work are: (i) the design of an indoor positioning system based on inertial measurements and the combination of RSS measurements from different sources (a smartphone and a smartwatch) using a GMM algorithm that improves the accuracy of the distance estimations; (ii) the identification and evaluation of the network deployment and network management issues (see Section 4) produced by the lack of control over the WiFi network that negatively affect the performance of positioning systems; and (iii) the experimental validation of the designed positioning system in a real scenario of 6000 $m^{2}$ without any control over the network and using commercial smartphones.

The rest of this paper is organized as follows: Section 2 introduces the architecture of the system. Section 3 details the algorithms employed to obtain the speed and the heading of the user from the inertial measurements. In Section 4, we describe the method used for computing the position estimation from the RSS measurements. The combination of inertial and RSS measurements is explained in Section 5, and Section 6 presents the experimental validation. Finally, the conclusions of the work are presented in Section 7.

## 2. System Architecture

Let us consider an arbitrary indoor area with a WiFi network formed by *N* anchor nodes with known positions $s_{i}={\left[\begin{array}{cc} x_{i} & y_{i} \end{array}\right]}^{T}$ for i=1,…,N defining a set,

(1) $$A=\{s_{1},s_{2},\ldots ,s_{N}\},$$

and a pedestrian user with unknown position defined by:

(2) $$m^{k}={\left[\begin{array}{cc} x^{k} & y^{k} \end{array}\right]}^{T},$$

where *x*, *y* are the respective Cartesian coordinates and *k* stands for the *k*-th time instant. Let us also consider that the user carries a smartphone (with an IMU) in the pocket. The IMU provides measurements from the accelerometer and gyroscope with a frequency of 100 Hz defining a set of measurements,

(3) $$I=\{{\mathrm{acc}}_{x},{\mathrm{acc}}_{y},{\mathrm{acc}}_{z},{\mathrm{gyr}}_{x},{\mathrm{gyr}}_{y},{\mathrm{gyr}}_{z}\}.$$

The smartphone periodically scans the WiFi channels every second and provides a set of measurements of the RSS from the anchor nodes, that is,

(4) $$R_{p}=\left\{{\mathrm{Rx}}_{1},{\mathrm{Rx}}_{2},...,{\mathrm{Rx}}_{N}\right\}.$$

We also consider that the user wears a smartwatch, which scans the WiFi channels every second and provides a set of RSS measurements, that is,

(5) $$R_{w}=\left\{{\mathrm{Rx}}_{1},{\mathrm{Rx}}_{2},\ldots ,{\mathrm{Rx}}_{N}\right\}.$$

**Figure 1:**
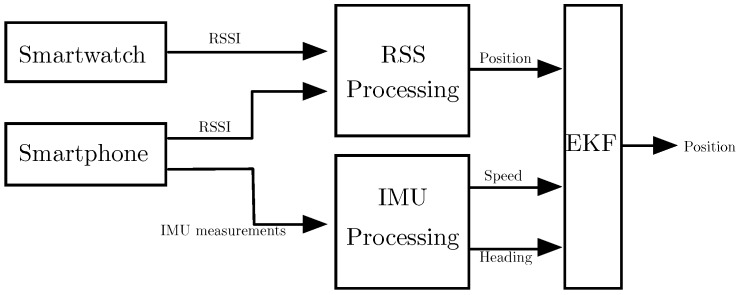
System architecture.

In this work, we combine all of the measurements provided by the smartphone and the smartwatch to compute an enhanced estimation of the user’s position. The architecture of the system (see Figure 1) is divided into three blocks: (i) the IMU processing block; (ii) the RSS processing block; and (iii) the filtering block. In the first block, we obtain the speed and heading of the user using the IMU readings of the smartphone. In the second block, the RSS measurements received by the smartphone and the smartwatch are combined and processed to obtain estimations of the user’s position. Finally, in the third block, the estimations obtained in the first two blocks are combined using an EKF that takes into account a constant velocity model.

## 3. IMU Processing Block

**Figure 2:**
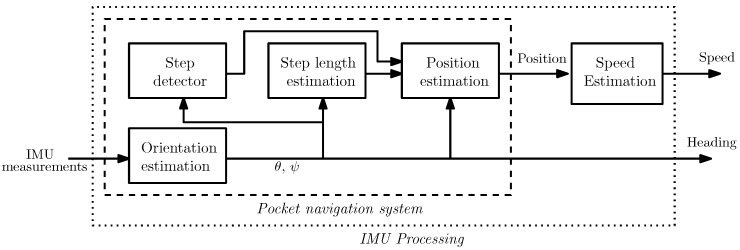
IMU processing block. The heading is estimated directly by the pocket navigation system. The speed is derived through the position estimate of the pocket navigation system.

The IMU processing block in Figure 1 processes the measurements of the inertial sensors embedded in the smartphone to estimate the user’s speed and heading. This is done in two steps as indicated in Figure 2. In the first step, the pocket navigation system uses the inertial measurements to estimate the user’s position and orientation defined by the Euler angles, i.e., roll (ϕ), pitch (*θ*) and heading (*ψ*), also known as yaw. In the second step, the position estimate is used to derive the speed of the user. The two parts of the inertial measurements’ processing are presented in the next sections.

Note that the pocket navigation system is based on the inertial measurements of a low-cost IMU embedded in a commercial smartphone running an Android OS. The smartphone is configured to provide measurements at a rate of 100 Hz. Unfortunately, the rate obtained is not exactly 100 Hz, and it will vary depending on the amount of processes running at the same time on the smartphone. This behavior impinges directly on the performance of the PDR algorithms because the IMU used is a low-cost IMU with higher biases, and the design measurement rate does not always match the operational measurement rate.

### 3.1. Pocket Navigation System

This section describes the pocket navigation system, which is presented in detail in [34] and the references therein. This system estimates the user’s position using inertial measurements collected with a smartphone placed in the user’s pocket.

The position is estimated through the step-length-and-heading-estimation approach. This recursive method estimates the position as follows:

(6) $$\begin{array}{ccc} x_{k} & = & x_{k-1}+S_{k}\times \cos\left(\psi_{k}\right), \end{array}$$

(7) $$\begin{array}{ccc} y_{k} & = & y_{k-1}+S_{k}\times \sin\left(\psi_{k}\right), \end{array}$$

where ($x_{k}$, $y_{k}$) is the x-y position of the user at time *k*, ($x_{k-1}$, $y_{k-1}$) is the x-y position of the user at the previous time k−1, $S_{k}$ is the step length at time *k* and $\psi_{k}$ is the heading estimate at time *k*.

The block diagram of the pocket navigation system is presented in the dashed box in Figure 2. The first subsystem is the orientation estimator. It uses turn rate and acceleration measurements to estimate the Euler angles, i.e., roll (ϕ), pitch (*θ*) and heading (*ψ*), of the user’s thigh.

The orientation estimation subsystem implements an unscented Kalman filter whose states are the Euler angles and the biases of the gyroscopes. The prediction stage of the filter integrates the turn rate measurements to obtain the Euler angles, whereas the biases are estimated with an auto-regressive model. The update stage of the filter corrects the orientation using the acceleration measurements. Further details can be found in [35].

The pitch (*θ*) and the heading (*ψ*) of the orientation estimation block are used in the next stages of the pocket navigation system. On the one hand, the heading is used for the position estimation as indicated by Equations (6) and (7). On the other hand, the pitch (*θ*) is used to detect step occurrences and estimate the step lengths, a method that was first proposed in [8]. For completeness, a brief overview is provided below.

**Figure 3:**
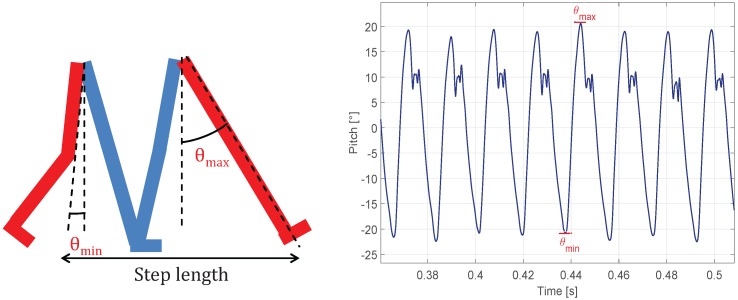
(**Left**) Maximum and minimum elongation of the red leg while walking; (**right**) Thigh pitch while walking. Each step can be detected by detecting each maximum of the pitch angle estimation (blue curve).

The left diagram of Figure 3 presents the maximum and the minimum elongation of the thigh during the walk. These are indicated by $\theta_{max}$ and $\theta_{min}$, respectively. Let us consider that the smartphone is on the thigh of the leg in red depicted in Figure 3. The maximum elongation corresponds to one step of the leg with the IMU, in this case, the leg with the smartphone. Thus, by detecting the maximums of the thigh pitch, step occurrences can be reliably detected [34]. Figure 3 also shows the evolution of the thigh pitch during eight steps.

Once a step is detected, its length needs to be estimated. The step length is estimated through the amplitude of the thigh pitch (Δθ). The latter is defined as the difference between the maximum and minimum elongation of the thigh with the smartphone; see Figure 3:

(8) $$\Delta \theta =\theta_{max}-\theta_{min},$$

The authors in [8] show that the relationship between the step length (*S*) and the amplitude of the thigh pitch (Δθ) can be represented by a first order model:

(9) S=a×Δθ+b,

where *a* and *b* are the parameters of the model that can be universal or can be estimated for each user.

By successively detecting maximums and minimums of the thigh pitch, not only steps can detected but also the step length can be estimated. This in combination with the heading estimated in the attitude estimation block, allows for a tracking of the user’s position.

### 3.2. Speed Estimation

The EKF of Figure 1 requires an estimation of the user’s speed. The latter is obtained by deriving the position estimate of the pocket navigation system in Figure 2. This is done as follows:

(10) $$v_{k}=\frac{p_{k}-p_{k-1}}{\Delta k},$$

where $v_{k}$ is the velocity at time *k*, $p_{k}$ and $p_{k-1}$ are the position estimates at times *k* and k−1, respectively, and Δk is the time increment between the position samples $p_{k}$ and $p_{k-1}$. The time increment, Δk, is calculated using the sampling time of each position estimate produced by the pocket navigation system. Then, we convert the speed to polar coordinates because the EKF uses a constant velocity model expressed in polar coordinates.

## 4. RSS Processing Block

This section details the algorithms employed to obtain position estimations from the RSS measurements. Traditionally, the position estimation is divided into two steps. First, the RSS is computed from an existing wireless network and transformed to distance estimations following a path loss model. Afterwards, the distance estimations are combined through a multilateration method to obtain the position estimation. The network selection is crucial for the design of an indoor positioning system. Among all possible network technologies that provide RSS measurements (WiFi, LTE, UWB, WSN, Bluetooth, etc.), we choose the WiFi technology because it has an outstanding advantage among all of the others: WiFi networks are currently deployed around the world in millions of buildings. Therefore, our system can be used in mass market applications without the need for any inversion in the network infrastructure. Unfortunately, WiFi networks are typically designed for communications and not for positioning applications. This fact generates some issues that must be amended in order to minimize the error of the positioning system. These issues can be divided into two groups: (i) network deployment issues; and (ii) network management issues.

### 4.1. Network Deployment Issues

The design of the network deployment for communication purposes tries to maximize the coverage area of the network with the minimum possible number of nodes. This minimization can be subjected to constraints as for example to assure a minimum QoS for the network in all of the area. These constraints can increase the number of anchor nodes, but generally, network designers are reluctant to increase the number of nodes as this increases the cost of the network infrastructure. This main design objective is contrary to the interest of positioning systems. The main issues follow:

- Reception from at least three anchor nodes: It is necessary to receive from at least three anchor nodes in order to obtain a 2D position estimation. Due to the low density of anchor nodes, there will be areas where the user will not receive from three of them. Note that this issue is magnified when the user only receives measurements from one anchor node.
- Distance to anchors: The mean distance between any anchor node and the mobile user is inversely proportional to the number of nodes. In combination with larger estimation errors at longer distances [36], the mean distance will affect the accuracy of the position estimations.
- Collinearity of anchor nodes: collinear anchors produce higher position estimation errors. The deployment of the nodes in a communication network does not take into account the collinearity of the nodes. Disregarding this issue will impinge on the performance of the positioning system.

### 4.2. Network Management Issues

The management of a communication network can respond to many different objectives, so it is difficult to analyze them in general. Notwithstanding, there are several configuration issues that can affect the performance of a positioning system:

- Transmitted power: Beyond the obvious relationship between the distance and the RSS, which is directly related to the transmitted power, the transmitted power also affects the coverage area of the anchor nodes, which can lead to the issues commented in the network deployment section (reception from at least three anchor nodes and distance to anchors).
- WiFi channel: Without controlling the network, the user is unaware of the transmission channel of the anchor nodes. Therefore, it has to scan all of the frequency channels in order to obtain the RSS, which increases the measurement time and reduces the number of measurements. Note that a reduction in the number of measurements means a reduction in the number of different anchor nodes received simultaneously.
- Beacon period of anchor nodes: Anchor nodes periodically send beacon signals, which are used to estimate the RSS. The periodicity of these signals affects directly the performance of the positioning system, as it determines the number of RSS estimations available for the user.
- Synchronization between anchor nodes: The synchronization between the anchor nodes is critical if we want to obtain at least three simultaneous RSS readings in order to compute a 2D position estimation.

The above issues can appear alone or in groups in an indoor positioning system. These issues are not commonly treated in the literature, as total control of the network is typically assumed, that is these issues are amended in the network configuration and design phase. However, without controlling the WiFi network, new ways of designing indoor positioning systems have to be considered. In this work, our approach is the introduction of a secondary receiver that ameliorates these issues. Focusing on our target of mass market applications, we will consider a smartwatch as the second receiver. These devices are nowadays increasing in popularity among consumers, and it is expected that they will be widely used in the near future.

The benefits of using a smartwatch as a secondary receiver in our system are mainly two: (i) an improvement in the accuracy of the distance estimations; and (ii) an increase in the number of measurements. On the one hand, the improvement in the accuracy of the distance estimations is achieved thanks to the combination of the RSS measurements from the two devices. By combining the RSS of two sources, the variability of the measurements is reduced. Note that we can consider that the noise of both measurements is statistically independent. Therefore, the accuracy of the distance estimation is increased. On the other hand, the increase in the number of measurements is produced by the fact that we have two devices independently scanning the WiFi channels, and therefore, the odds of receiving more measurements are increased.

**Figure 4:**
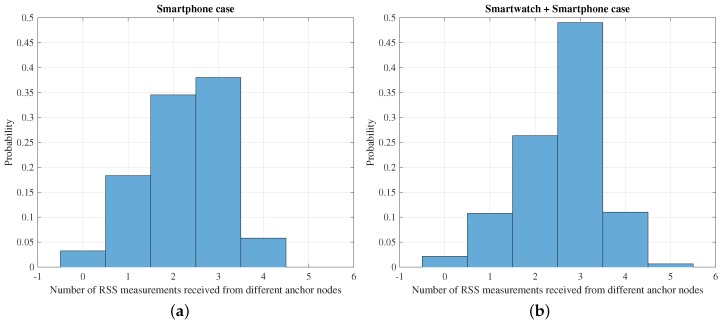
Normalized histograms of the number of RSS measurements received from different anchor nodes at each time instant: (**a**) case of only using the smartphone for the measurements (**b**) case of using the smartphone and the smartwatch.

Figure 4 shows the normalized histogram of the number of RSS measurements from different anchor nodes received by the system at each time instant. In Case A, only the smartphone is used to obtain the measurements, and in Case B, the smartphone and smartwatch measurements are used. An increase of the number of times that the system has received from three or more different anchor nodes can be observed, which results in an increase in the accuracy of the position estimation, as is shown in Section 6. Particularly, in Case A, the number of times that the system has received from more than three anchor nodes represents 44% of times, whereas in Case B, this number rises up to 61%.

### 4.3. Distance Estimation

Typically, the estimation of distance from the RSSI uses the log-distance path loss model [9], that is,

(11) $$P=P_{1m}-10\alpha \log_{10}d-\gamma ,$$

where *P* is the received power, $P_{1m}$ is the received power at one meter from the transmitter, *α* is the path-loss exponent, *d* is the distance and $\gamma ∼N\left(0,\sigma_{\gamma }^{2}\right)$ models the shadowing effects. Considering this model, the RSS follows a Gaussian distribution:

(12) $$P∼N\left(P_{1m}-10\alpha \log_{10}d,\sigma_{\gamma }^{2}\right),$$

However, this model was developed for the single receiver case and does not take into account the scenario proposed in this work where the user carries two different receivers. As an alternative, in this work, we propose the use of Gaussian mixture models (GMM). The idea behind the mixture models is to obtain a new distribution from a linear combination of known distributions, Gaussian in the case of GMM. By using a sufficient number of Gaussians and by adjusting their means and covariances, as well as the coefficients in the linear combination, almost any continuous density can be approximated to arbitrary accuracy [37]. We consider a superposition of *K* Gaussian distributions of the form,

(13) $$p\left(X\right)=\sum_{k=1}^{K}\pi_{k}N\left(X|\mu_{k},\Sigma_{k}\right),$$

where $\pi_{k}$ are the so-called mixing coefficients and $\mu_{k}$ and $\Sigma_{k}$ are, respectively, the mean and covariance of the Gaussian densities used (also known as components of the mixture). In order to use the GMM in the distance estimation, we need to compute the parameters of the model, i.e., $\pi_{k}$, $\mu_{k}$ and $\Sigma_{k}$, from a set of observations obtained in a calibration phase. Lets us denote *X* as the set of *N* observations obtained from the calibration phase, that is a set of *N* vectors $\left[\begin{array}{ccc} R_{p}, & R_{w}, & d \end{array}\right]$, where $R_{p}$ are the RSS received by the smartphone, $R_{w}$ the RSS received by the smartwatch and *d* the real distance from the user to the anchor node. Assuming that the data points are drawn independently from the distribution, the log likelihood function is given by,

(14) $$\ln p\left(X|\pi_{k},\mu_{k},\Sigma_{k}\right)=\sum_{n=1}^{N}\ln\left(\sum_{k=1}^{K}\pi_{k}N\left(x_{n}|\mu_{k},\Sigma_{k}\right)\right).$$

We compute the parameters of the model as the values that maximize the log likelihood function. The maximization of the log likelihood function of a GMM is a complex problem and does not have a closed solution. Traditionally, the maximization problem is solved using the expectation maximization (EM) algorithm [38]. The EM algorithm is an iterative method that maximizes the log likelihood function in each iteration following a two-step procedure: the expectation step (E-step) and the maximization step (M-step). [37]. In the E-step, we compute the so-called responsibilities $\gamma \left(z_{n,k}\right)$ from the current estimation of the parameters, that is,

(15) $$\gamma \left(z_{n,k}\right)=\frac{\pi_{k}N\left(x_{n}|\mu_{k},\Sigma_{k}\right)}{\sum_{j=1}^{K}\pi_{j}N\left(x_{n}|\mu_{j},\Sigma_{j}\right)},$$

while in the M-step, we re-estimate the parameters using the computed responsibilities, that is,

(16) $$\mu_{k}^{new}=\frac{1}{N_{k}}\sum_{n=1}^{N}\gamma \left(z_{n,k}\right)x_{n},$$

(17) $$\sigma_{k}^{new}=\frac{1}{N_{k}}\sum_{n=1}^{N}\gamma \left(z_{n,k}\right)\left(x_{n}-\mu_{k}^{new}\right){\left(x_{n}-\mu_{k}^{new}\right)}^{T},$$

(18) $$\pi_{k}^{new}=\frac{N_{k}}{N},$$

where:

(19) $$N_{k}=\sum_{n=1}^{N}\gamma \left(z_{n,k}\right).$$

These two steps are iteratively repeated until the increment of the log likelihood function in the current iteration is below a convergence threshold.

As previously stated, in this work, we do not have any control of the wireless network. This will produce a lack of synchronization between the RSS measurements of the receivers, and therefore, we have to consider different situations:

- Case A: In this case, we obtain a measurement of the RSS from a specific anchor node in both receivers at a quasi-simultaneous time. Then, we can compute the estimated distance using the RSS obtained by both devices. Therefore, we define a vector of observation $X=\left[\begin{array}{ccc} RSS_{p}, & RSS_{w}, & d \end{array}\right]$.
- Case B: In this case, we receive RSS measurements only from the smartphone. Then, we define the observation vector as $X=\left[\begin{array}{cc} RSS_{p}, & d \end{array}\right]$.
- Case C: This is similar to Case B, but we receive RSS measurements from the smartwatch instead of the smartphone; the observation vector is $X=\left[\begin{array}{cc} RSS_{w}, & d \end{array}\right]$.

We use a different GMM for each one of the three cases, that is we estimate the parameters $\pi_{k}$, $\mu_{k}$ and $\Sigma_{k}$ for the log likelihood function of each one of the cases. The difference between the models is the vector of observations *X*. Once we have estimated the parameters of the models using the data obtained in the calibration phase and the EM algorithm, we estimate the distance solving the following maximization problem:

(20) $$\hat{d}=\max_{\hat{d}}\ln p\left(X|\pi_{k},\mu_{k},\Sigma_{k}\right),$$

where the maximization of the log likelihood function is done with respect to the distance because it is the only unknown variable in the vector of observation *X*; the $RSS_{p}$ and $RSS_{w}$ variables are known as they are the RSS measurements received from the smartphone and the smartwatch.

### 4.4. Position Computation

As stated in the beginning of this section, the estimation of position based on RSS measurements is a two-step procedure. Once the distances to the anchor nodes are estimated, we must combine them to obtain the position of the user. In this work, we use a weighted least squares algorithm (WLS). The WLS estimates the user position in order to minimize the overall squared error of the distance measurements. In other words, the position is computed as the solution to the following minimization problem [39],

(21) $$\min_{{\hat{m}}^{k}}\sum_{i\in D}\omega_{i}{\left({\hat{d}}_{i}-∥s_{i}-{\hat{m}}^{k}∥\right)}^{2},$$

where D is the set of distance measurements available at time instant *k* and $\omega_{i}=\frac{1}{{\left({\hat{d}}_{i}\right)}^{2}}$ are the weights of the algorithm [40]. Note that due to the logarithmic relationship between the RSS and the distance, the accuracy of the estimations depends on the distance to be estimated itself [36], and hence, it is meaningful to assign different weights to estimations with different accuracies [41]. The problem in Equation (21) can be solved in an iterative way following a gradient descent approach.

## 5. Filtering Block

The third block of the system architecture is the filtering block. Here, we combine the estimations of the position, speed and heading of the user into a single filter that outputs an enhanced estimation of the user’s position. In this work, we have employed the EKF, which is an extension of the KF designed to cope with non-linear models [42]. Although the PF is a common option in the related works due to its ability to adapt to non-Gaussian measurement errors, the use of a PF increases the computational complexity of the system. Our objective is to design a system for mass market applications; thus, the overall computational complexity and its associated battery consumption must be reduced. For this reason, we choose the EKF algorithm, which presents a good balance between positioning accuracy and computational complexity.

Let us model the state of a person in a two-dimensional space by means of its position and velocity,

(22) $$x^{k}={\left[\begin{array}{cccc} x^{k} & y^{k} & V^{k} & \psi^{k} \end{array}\right]}^{T},$$

where $x^{k},y^{k}$ represent the position in Cartesian coordinates, $V^{k}$ is the speed and $\psi^{k}$ the heading of the user. The EKF algorithm is divided into two steps: (i) the prediction step; and (ii) the correction step. In the prediction step, the a priori estimation of the state vector ${\hat{x}}_{-}^{k+1}$ and the state covariance $P_{-}^{k+1}$ are updated using the kinematic model,

(23) $${\hat{x}}_{-}^{k+1}=F\left(\psi^{k}\right){\hat{x}}^{k},$$

(24) $$P_{-}^{k+1}=M^{k}P^{k}{\left(M^{k}\right)}^{T}+Q,$$

where *k* is the time step, $F\left(\psi^{k-1}\right)$ is the transition matrix, $v^{k}$ is a zero mean Gaussian noise with covariance matrix Q and $M^{k}$ is the Jacobian matrix of the partial derivatives of the model function $F\left(\psi^{k}\right)x^{k}$ with respect to $x^{k}$. The transition matrix is defined following the constant velocity model with the velocity represented in polar coordinates, that is,

(25) $$F\left(\psi^{k}\right)=\left[\begin{array}{cccc} 1 & 0 & T\cos\psi^{k} & 0\\ 0 & 1 & T\sin\psi^{k} & 0\\ 0 & 0 & 1 & 0\\ 0 & 0 & 0 & 1 \end{array}\right],$$

where *T* is the time period between measurements. In the second step of the method, i.e., the correction step, the previous predictions are corrected thanks to the measurements and the so-called Kalman gain $K^{k}$ [43], that is,

(26) $$K^{k+1}=P_{-}^{k+1}H^{T}{\left(HP_{-}^{k+1}H^{T}+R^{k+1}\right)}^{-1},$$

(27) $${\hat{x}}^{k+1}={\hat{x}}_{-}^{k+1}+K^{k+1}\left(z^{k+1}-H{\hat{x}}_{-}^{k+1}\right),$$

(28) $$P^{k+1}=\left(I-K^{k+1}H\right)P_{-}^{k+1}.$$

where H is the measurement matrix, the identity matrix in our case, $z^{k+1}$ is the measurement vector and R is the measurement noise covariance matrix.

The EKF exploits the statistics of the measurements in order to produce an enhanced estimation of the state vector. These statistics are considered to be known by the designer and are introduced to the filter through the configuration of the covariance matrices Q and R. In this work, we assume that the state variables and measurement variables are independent, and therefore, we configure their covariance matrices as diagonal matrices. The values of the variances are manually configured based on our experimental results. The specific values used are:

(29) $$Q=diag\left(\left[\begin{array}{cccc} 0.5 & 0.5 & 3 & 3 \end{array}\right]\right),$$

(30) $$R=diag\left(\left[\begin{array}{cccc} 75 & 75 & 0.5 & 0.05 \end{array}\right]\right),$$

The following section validates the performance of the designed pedestrian tracking system in a real environment.

## 6. Experimental Validation

In this section, we test the performance of the indoor positioning system designed. To evaluate the performance, we have used a Motorola Moto G2 LTE smartphone and a Nexus 5 smartphone, which will take the roll of the smartwatch once attached to the wrist of the user. Both smartphones include a 6-DoF IMU with an accelerometer and a gyroscope. The sensors are sampled with a frequency of 100 Hz with our own designed Android application that also scans the WiFi channels to obtain the RSS measurements. As stated in Section 3, the obtained frequency is not always 100 Hz, and our system has to adapt to small changes in the measurement rate. The system updates the estimation of the user’s position every second using the inertial and RSS measurements received since the last estimation update.

**Figure 5:**
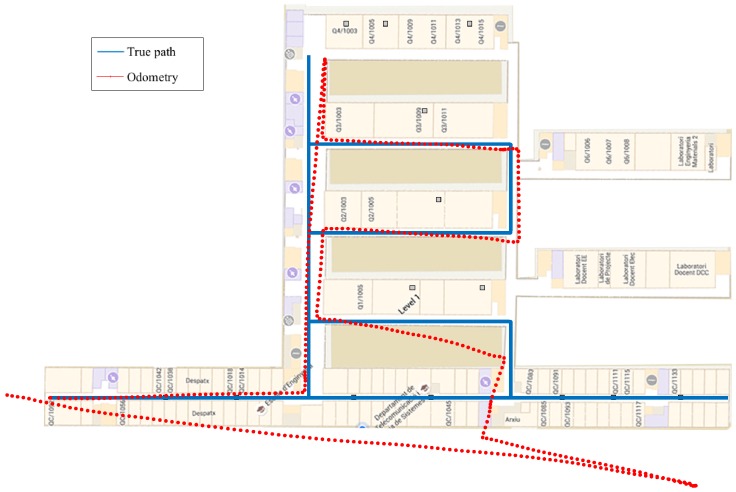
Path 1 and odometry obtained with the pocket navigation system.

The scenario of validation is the first floor of the Engineering School at the Universitat Autònoma de Barcelona (see Figure 5). The scenario is an area of approximately $6000\;m^{2}$ covered with 14 WiFi access points that will act as the anchor nodes of the system, that is an anchor node every $428.5\;m^{2}$. Note that there is no control about the configuration nor the placement of the anchor nodes as far as we are using the available WiFi network at the building. Note also that the distribution of the anchor nodes is not uniform around the area, and there are zones far from any anchor nodes, zones with high density of anchor nodes and zones with collinear anchors nodes. This is the reason for choosing this scenario; the selected scenario presents all of the typical issues of a communication network described in Section 4.

**Figure 6:**
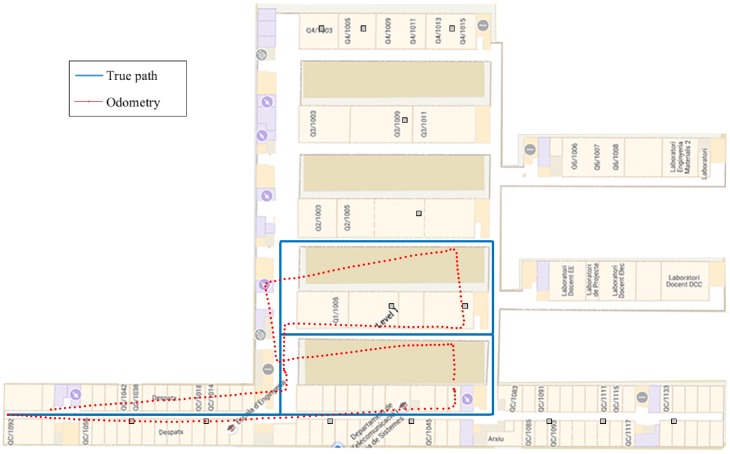
Path 2 and odometry obtained with the pocket navigation system.

**Figure 7:**
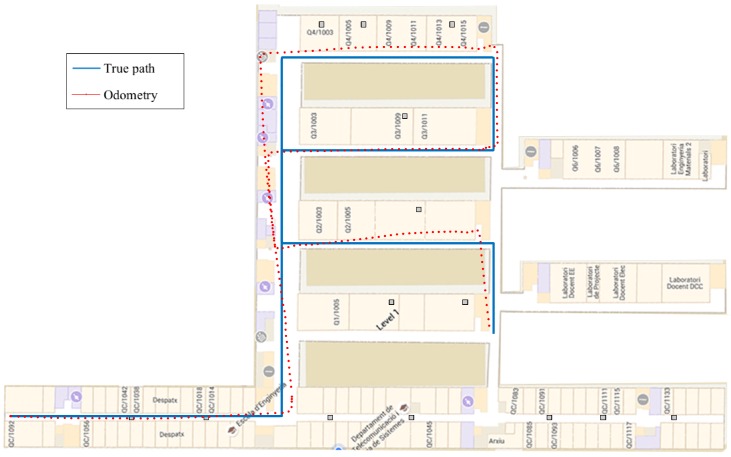
Path 3 and odometry obtained with the pocket navigation system.

In order to evaluate the indoor positioning system, we have considered three different paths. Figure 5 depicts the trajectory of Path 1 with a length of 620m. The second path, i.e., Path 2, is shown in Figure 6, and it has a length of 420m. Finally, Path 3 is shown in Figure 7, and it has a length of 338m.

During the experimental validation, we compare five different methods: (i) a step-length-and-heading-estimation algorithm based on the inertial measurements from the smartphone; (ii) a WLS approach based on the distance estimations obtained from the RSS measurements of the smartphone; (iii) an EKF that combines the estimations of the previous WLS (only based on the smartphone) with the inertial measurements; (iv) a WLS based on the distance estimation obtained from the combination of the RSS measurements of the smartphone and the smartwatch; and (v) an EKF that combines the inertial measurements with the WLS based on the smart combination of the smartphone and smartwatch. In the following, these methods are referred to as IMU, WLS-phone, EKF-phone, WLS-smart and EKF-smart, respectively.

In order to evaluate the performance of the different methods, we compute the real path of the user using a series of landmarks deployed around the building. We ask the user of the system to press a button every time that he/she goes near a landmark. Then, the times are stored, and the real path is calculated by assuming a constant speed of the user between two landmarks. With the appropriate number of landmarks, the resultant error of the obtained real path can be considered negligible.

Table 1 shows the results obtained during the experimental validation in terms of the RMSE. We have selected the paths in order to be representative of a real scenario. For this reason, we have selected two paths where the IMU has a considerable drift (Path 1 and Path 2) and one path where the drift of the IMU is small (Path 3). The odometry of the selected paths is shown in Figure 5, Figure 6 and Figure 7. Results show the improvement in the positioning accuracy due to the use of a second mobile receiver. In particular, the improvement of the WLS-smart over the WLS-phone is around 30% in the case of Path 1, 23% in Path 2 and 37.5% in Path 3. For the EKF-smart system, the improvement over the EKF-phone is around 35% in the case of Path 1, 19% in the case of Path 2 and 22% in Path 3. If we consider the improvement of the EKF-smart system compared with the IMU system, we obtain an improvement of 56% for Path 1, 35% for Path 2 and 12.5% for Path 3. The comparison between the WLS-smart method and the WLS-phone method gives us an idea about the improvement produced by the use of a second receiver in the system. The increment in the accuracy of the estimation is produced by two main factors. First, the combination of the RSS from the smartphone and the smartwatch results in an increment in the accuracy of the distance estimation. Second, the use of two receivers increases the number of measurements available. Specifically, the increase in the number of measurements is 35% in Path 1, 25% in Path 2 and 28% in Path 3. These results confirm that the use of a second receiver ameliorates the problems of RSS-based methods implemented with third party WiFi networks stated in Section 4. Similarly, the comparison between the IMU method and the smart-EKF shows the benefits of combining inertial measurements with RSS measurements because the PDR system employed obtains high accurate position estimations in the short term, but deviates with time. Contrarily, the accuracy of the RSS position estimations is lower, but the estimation of position is time invariant. A special case of interest appears in Path 3 when we compare the accuracy of the EKF-phone method with the IMU method. In this case, the combination of inertial and RSS measurements from the smartphone has worse performance than the use of only the IMU measurements. This effect is produced by the general configuration of the parameters of the EKF (see Section 5) as the configuration is fixed for all of the cases that the EKF cannot adapt to the increase in the accuracy of the IMU measurements, increasing the importance of the IMU measurements in the output of the EKF. Instead, the IMU measurements are treated in general as in the other cases, and the output does not improve the accuracy of the single IMU system. Note that this effect is not present in the EKF-smart system because the combination of RSS measurements from two sources increases the accuracy of the prior position estimations (see WLS-smart), and in this case, the difference between the accuracy of the IMU and RSS measurements is similar for the three paths; therefore, the general configuration works well for all of them, and the EKF-smart system always outperforms the accuracy of the other systems.

**Figure 8:**
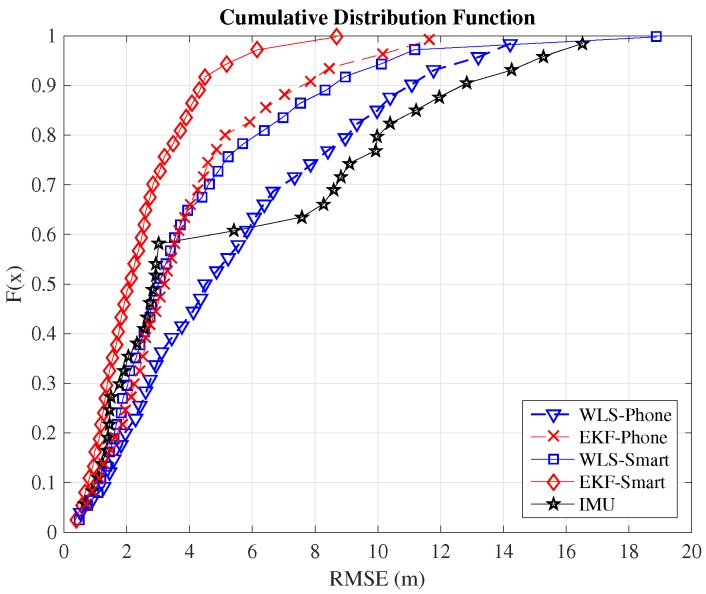
CDF of the positioning estimation error in Path 1.

**Figure 9:**
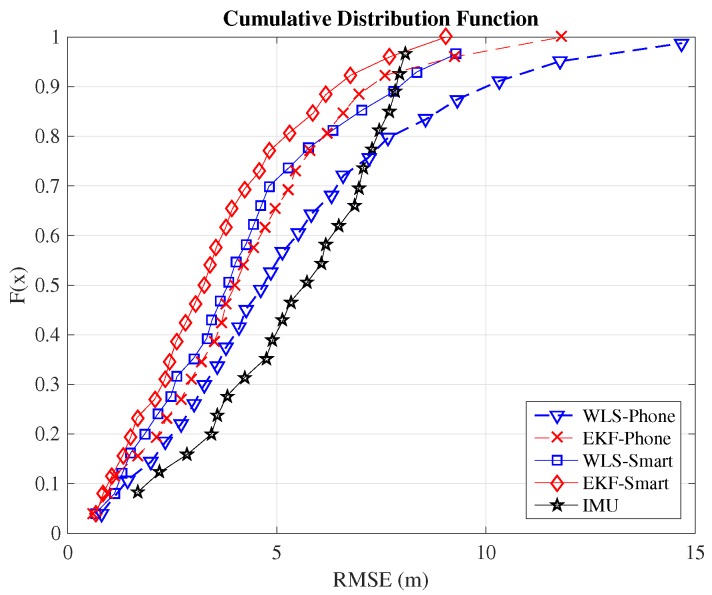
CDF of the positioning estimation error in Path 2.

**Figure 10:**
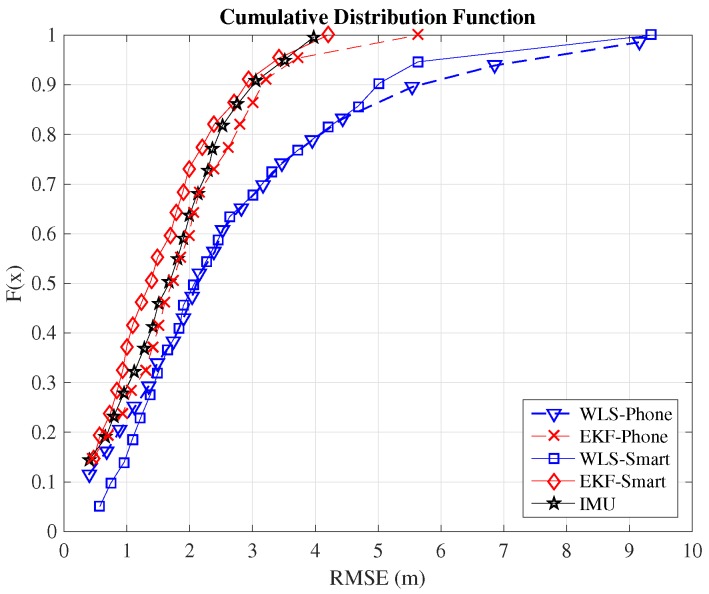
CDF of the positioning estimation error in Path 3.

Although the typical metric for comparison between indoor positioning systems is the RMSE, we include here also the cumulative distribution function (CDF) because it provides additional information. In order to compare the positioning systems employed in this work, we will consider the value of the RMSE when the CDF equals 0.9. This means that the error committed by the algorithm is below this threshold value in 90% of the cases. Table 2 summarizes the 90 percentiles of the validated systems. The complete CDFs are shown in Figure 8 for the case of Path 1, Figure 9 for the case of Path 2 and Figure 10 for Path 3. From the figures, we can observe how the EKF-smart outperforms all of the other systems. It can also be observed how the error committed by our system is almost always below 2.9m, 4.4m and 6.3m, which can be considered a good accuracy taking into account the area of the scenario, which is $6000\;m^{2}$. If we compare the accuracy of our system with other indoor positioning systems based on smartphones, we can see that our system outperforms similar systems based on hybrid measurements [25,26,28] and obtains accuracies similar to the ones obtained by hybrid systems that include additional map information for the sensor fusion [31,32,33].

The experimental validation shows the benefits of introducing a smartwatch in an indoor positioning system based on a smartphone. The positioning accuracy obtained from the RSS measurements of the WiFi network is increased, and therefore, the overall solution including also the inertial measurements shows better performance. In fact, in the scenarios tested, the increment in accuracy from the initial PDR algorithm to the EKF-smart methods goes from 12.5% up to 56%. Furthermore, the system has been designed for being used in conjunction with a third party WiFi network, and the experimental validation has proven the adaptability of our system and its ability to obtain accurate position estimations.

## 7. Conclusions

This work aims at developing an indoor positioning system that can be applied to mass market applications. For this reason, we have selected a smartphone and a smartwatch as the devices that the user will carry in order to obtain his/her position. The designed system combines the inertial measurements of the smartphone placed in the pocket of the user with the RSS measurements from a WiFi network obtained by the smartphone and the smartwatch. On the one hand, the position estimation based on the inertial measurements follows a step-length-and-heading-estimation approach that uses the pitch angle of the thigh to detect steps and the amplitude of the thigh pitch to estimate the step length. The inertial measurements are obtained from a commercial smartphone with an embedded low-cost IMU. The designed PDR system is able to adapt to small changes in the measurement rate of the inertial sensors. On the other hand, the position estimation based on the RSS of the WiFi network is computed using a GMM that combines the measurements of the two receivers to obtain an enhanced distance estimation. We also analyze the challenges of using an external WiFi network designed for communication purposes and without any control over the network configuration. We have demonstrated how the use of a smartwatch ameliorates these issues improving the overall accuracy of the system. The combination of the inertial and RSS measurements has been done using an extended Kalman filter with a constant velocity kinematic model. The system has been experimentally validated in an scenario with an area of $6000\;m^{2}$, and the results show that the use of two RSS receivers in conjunction with the inertial measurement of a smartphone placed in the pocket of the user can improve the accuracy of the position estimation up to 56%.

## Figures and Tables

**Table 1:** Results of the experimental validation in terms of RMSE. WLS, weighted least squares.

	Smartphone				Smartphone & Smartwatch	
	IMU	WLS-Phone	EKF-Phone		WLS-Smart	EKF-Smart
Path 1	5.4	5.6	3.7		3.9	2.4
Path 2	5.2	5.3	4.2		4.1	3.4
Path 3	1.6	4	1.8		2.5	1.4

**Table 2:** Results of the experimental validation in terms of the 90 percentile.

	Smartphone				Smartphone & Smartwatch	
	IMU	WLS-Phone	EKF-Phone		WLS-Smart	EKF-Smart
Path 1	12.8	11	7.8		8.5	4.4
Path 2	7.9	10	7.3		8	6.3
Path 3	3	5.6	3.1		5	2.9
